# Preparedness, Identification and Care of COVID-19 Cases by Front Line Health Workers in Selected Health Facilities in Mbale District Uganda: A Cross-Sectional Study

**DOI:** 10.24248/eahrj.v5i2.665

**Published:** 2021-11-15

**Authors:** Naziru Rashid, Aisha Nazziwa, Nicholas Nanyeenya, Nabukeera Madinah, Kamada Lwere

**Affiliations:** a Department of Community Medicine and Public Health, Habib Medical School Islamic University in Uganda; b Uganda National Health Laboratory Services Ministry of Health Uganda; c Faculty of Management Studies Islamic University in Uganda

## Abstract

**Introduction:**

The nature of work of Health care professionals exposes them to high risks of contracting COVID-19 and spreading it among themselves, to their patients and subsequently to the general community. Thus, it is essential that frontline health workers are equipped with both material and knowledge to enable them accurately suspect, detect, isolate, and manage COVID-19 cases. Findings have indicated a high prevalence of COVID-19 infections among front-line health workers. The Current Study assessed preparedness, identification, and care of COVID-19 Cases by frontline health workers in selected health facilities in Mbale District.

**Methodology:**

Across sectional survey was used to collect quantitative data using Google forms, An online platform for data collection. Data was collected from 189 frontline health workers in both government and private Health facilities in Mbale District between April and August 2020. Data was analysed using Statistical Package for the Social Sciences (SPSS) version 20.

**Findings:**

The study found that a good proportion of frontline health workers can identify cases by symptom and case definitions as probable case 113/189(59.8%), suspected case 60/189(36%) and confirmed case 22/189 (11.6%). There were generally low levels of preparedness in terms of initial service care being offered with the highest being 53/189(28.2%) and 50/189(26.4%) for facilities that had places for isolation and those with intravenous fluids respectively and the least was being able to offer oxygen and Intensive Care Services at 43/189(22.0%) and 20/189(10.3%) respectively.

**Conclusion and recommendations:**

There's a need to ensure a continuous supply of PPEs and IPC materials to health facilities. CPD programs are essential in equipping Health workers with up-to-date information on COVID-19 Case Management. Facilities should be supported to setup isolation facilities at all levels, both permanent and temporary. Provision of Face masks to health workers should be prioritised and hand washing facilities should be installed at every serving point.

## BACKGROUND

Coronavirus disease (COVID-19) is an infectious respiratory disease caused by the Severe Acute Respiratory Syndrome Corona Virus 2 (SARSCoV-2 virus).^1,2^ Evidence has shown that the virus is spread through birds and bats, with humans being particularly vulnerable to infections due to the virus.^3^ Among humans, it is transmitted through droplets of saliva, discharge from nose coughs, or sneezes from an infected person.^1,4,5^

COVID-19 infections are characterised by symptoms ranging from mild to severe cough, flu, fever, general body pains, difficulties in breathing, septic shock, multi-organ failure and death respectively. The risk of severity is high among the elderly and people with other comorbidities.^6–8^

The World Health Organization (WHO) reported cases of Pneumonia Like illness of unknown cause on the 31st December 2019.^5,9^ The Causative Pathogen of this illness was later identified as Severe Acute Respiratory Syndrome Corona Virus-2 (SARSCoV-2) and was declared a global pandemic known as Corona Virus disease 2019 (COVID-19) on the 11^th^ March 2020 by WHO.^2,3^

The Pandemic has since spread globally in many countries around the world. As of 2^nd^ June 2021, there are more than 170 million Confirmed cases of COVID-19 globally, about 3.5 million Cases in Africa and 47,761 cases from Uganda. Globally 3.5 Million deaths have been reported as a result of COVID-19 of which 362 are from Uganda as of June 2^nd^ 2021.^10^

Front line health workers like; doctors, Clinical officers, nurses, and other Paramedics are among the most at-risk populations for contracting COVID-19 and as of 20^th^ January 2021, 1,873 health workers had been infected with the COVID-19 Virus with many Deaths being reported Globally. As of June 2021, Uganda's population is approximately 41 million people, and these reside mostly in urban centres and towns. This high population density in towns and urban centres makes it difficult to observe social distancing and practice good hygienic measures like hand washing especially in congested and busy working places like markets, shopping malls and public transport stages.

In response to the Pandemic, the Ugandan Government through the Ministry of Health (MoH) put measures and guidelines. These included; the closure of academic and religious institutions, offices, markets and trading malls, banning of both public and private transport systems as well as instituting a total country lockdown as a way of preventing and controlling the spread of COVID-19.^11,12^ Control measures at an individual personal level were also emphasised. These included; regular hand washing with water and soap, hand sanitisation, avoiding touching of the mouth, nose, and eyes, coughing and sneezing in elbows or paper tissues, wearing face masks and avoiding crowds by staying home.^4,5,13^

The nature of work of Health care professionals exposes them to high risks of contracting COVID-19 and spreading it among themselves, to their patients and subsequently to the general community. It is therefore essential that frontline health workers can accurately suspect, detect, isolate, and manage COVID-19 cases. This is very crucial in controlling and minimising the spread of the infection among health workers and to their patients. Studies conducted in Mumbai India and southwest Iran indicated high prevalence of COVID-19 infections among the front line health workers.^14,15^ In this study, we assessed the ability to identify a COVID-19 Case, take appropriate actions and the level of preparedness of Health Facilities to handle COVID-19 in Mbale Region, Uganda.

The findings of this study will help authorities in designing appropriate case definition and case management guidelines for all health workers at different levels, help in designing appropriate Continuing Professional Development (CPD) strategies for health workers at various stages

## METHODOLOGY

### Study Setting and Rationale

The study was carried out in selected health facilities in the Mbale district.

Mbale district is in the Eastern region of Uganda in East Africa, approximately 225km (140miles) North East of the capital Kampala. By 2019, the district's population was estimated at 568,000 people, 52.3% being females.^2^ The main economic activities in Mbale District are farming and trade (business). Mbale district is located along the highway connecting Kenya to South Sudan and the Democratic Republic of Congo through Uganda. The town attracts many people from the neighbouring communities and districts who come in for trade and other office jobs. In addition, there are several social-cultural and religious activities that take place in the district. Activities like the region's traditional dance (Kadodi) increase the high risk of spread of communicable diseases like COVID-19 in the community. Mbale District registered the first COVID-19 Deaths in Uganda.^1,16^

The big community in Mbale seeks medical services in several private, Faith-based, and public/government health facilities within the district. This puts the frontline health workers at risk of contracting the disease in case they fail to quickly identify probable COVID-19 cases. The district has 12 Government dispensaries (Level II), 17 sub-county health centres (Level III), and 4 health centres (level IV) at sub-district with 2 hospitals. It also has 4 private/NGO dispensaries, 7 health centres (III), and no private hospital. There is a government Regional Referral Hospital with 332 beds.^16,17^

### Study Design and Rationale

The study design was a descriptive cross-sectional survey employing quantitative methods of data collection.

### Study Population

The target population was frontline health workers involved in day to day running of health facilities including private, Private Not-For-Profit (PNFP) and public health facilities in the Mbale district. Frontline health workers included nurses, midwives, laboratory personnel, intern students, doctors, and clinicians among others. This is the team that comes into fast contact with patients seeking health services and thus the first contact with possible COVID-19 cases.

### Sample Size Determination

The sample size for this study was calculated using Kish Leslie (1964) formula for single proportion.

$$n=\frac{z^{2}pq}{d^{2}}$$

Where: n is the sample size

*d* is the precision., A precision of 5% was used.

*z* is the Standard Deviation corresponding to 95% and Confidence Interval of 1.96.

*p* is the Prevalence.

### Sampling Procedure

Simple random sampling was used to select health workers in private, private not-for-profit and public facilities in the Mbale district. An online survey questionnaire was the shared with staff through emails, WhatsApp, and Facebook messenger. Participants were requested to consent their participation in the study. The questionnaires were filled and submitted online.

### Inclusion Criteria

All frontline health workers in private, private not-forprofit and public facilities in the Mbale district involved in day-to-day patient care who had access to the internet via computers or mobile phones and consented to participate in the study were included.

### Exclusion Criteria

Health facility staff members who are not directly involved in patient care (including but not limited to administrators, store managers, medical record personnel among others) were excluded. Health workers who did not consented to participate in the study were also excluded.

### Research Instruments

Data was collected using structured Self-administered questionnaires sent via either emails, WhatsApp and/or Facebook messenger. The questionnaires were filled online, and data was captured automatically. The Questionnaire covered the following parameters: Demographics, Case Definitions and identification, Immediate Actions that were taken, Availability of Emergency service facilities and presence or absence of CPD programs in Health Facilities.

Case Definitions and Management were based on WHO and Ugandan government's through the Ministry of Health Guidelines for COVID-19 Case Management. The Questionnaire was first shared among the health workers of (Islamic University in Uganda (IUIU) Health centre for validation. All concerns raised were corrected.

### Data Management

After thorough checking, editing, tallying, and coding of the data from the filled questionnaires, data was entered and stored in Microsoft excel for analysis.

### Quantitative Data Analysis

Data was cleaned and analysed using Statistical Package for the Social Sciences (SPSS) (IBM Corp. Released 2011. IBM SPSS Statistics for Windows, Version 20.0. Armonk, NY: IBM Corp.)

### Ethical Considerations

**Anonymity:** Participants' identification information was not included anywhere in the data collection tool and thus was not captured.

**Confidentiality:** The Health Facilities' names where the health workers are working were also not mentioned (for confidentiality issues). These were identified by their level of service (i.e., health centre levels II, III, IV and hospitals) and whether government, private or private not for profit. This was intended to maintain confidentiality of the individual health facilities.

**Informed consent:** All participants were requested to sign an informed consent before participating in the study. This was done before the filling of the online questionnaire (Google forms). Participants were informed of their right to withdraw from the study without consequences at any stage of the study.

Permission to conduct the study was sought for from the Research Coordination Committee (RCC) of the Islamic University in Uganda. Permission was granted with reference number RCC/FHS/20/003

## RESULTS

### Demographic Findings

The study consisted of 189 health workers (participants). Of the 189 health workers, 65/189 (34.4%) were females and 123/189 (65.1%) were males. 1 preferred not to say.

86/189 (45.5%) of the respondents were between 20 and 29 years of age, 76/189 (40.2%) were between 30 and 39 years, 19/189 (10.1%) were between 40 and 49 years, and 8/189 (4.2%) were above 50 years. 79/189 (35.9%) of the participants had Bachelor's degrees, 19/189 -(8.6%) had certificates, 58/189 (26.4%) had Diplomas, 29/189 (13.2%) had Master's degree and 4/189 (1.8%) had other Post Graduate qualifications.

33(17.36%) were Nursing and Medical Students, 14(7.37%) were Enrolled Nurses and Midwives, 20(10.52%) were Nursing officers, 36(18.94%) were Clinical officers, 32(16.84%) were Laboratory staff, 29(15.26%) were Medical Doctors, 5(2.7%) were specialists, 18(9.8%) were public health officers and 3(1.57%) were categorised as other Paramedics.

33.9% the participants were from Hospitals, 11.1% were from Health centre IVs and 54% were from Health Centre IIIs and IIs.

Lastly, 43.8% (83) were from Government Health Facilities, 24.9% (47) were from Private Not for Profit Health Facilities, 28.1% (53) were from Private Health Facilities and 3.2% did not mention.

### Identification of COVID-19 Suspect or confirmed Case and the appropriate Immediate Action

To assess the ability to suspect and detect COVID-19 cases and the ability to take appropriate response, 5 questions were asked. These included the definitions of suspected probable and confirmed COVID-19 case and identification of COVID-19 respiratory symptoms. The results were as follows: 60/189 (36%) of participants could tell the appropriate definitions of a suspected case, 113/189 (59.8%) a probable case and 165/189 (87.3%) could tell a confirmed case. 22/189 (11.6%) were able to identify respiratory symptoms and signs that define COVID-19. Only 7/189 (3.7%) could take the appropriate immediate actions in case of suspected COVID-19 cases. The results are summarised in Table 1.

**TABLE 1:** Identification of COVID-19 Case and the appropriate Immediate Action taken

Questions for Identification of COVID-19 Case and Responses	N=189	%
A suspected case of COVID-19 is one with the following signs and symptoms. (tick all that apply)		
**A patient with acute respiratory illness.** **Fever above 37.5 plus at least one symptom/sign of respiratory disease.** **History of travel to a or residence of region reporting community transmission of COVID-19 disease during the last 14 days.** **A patient with an acute respiratory illness and has been in contact with confirmed or probable COVID-19 case** A patient with respiratory signs and symptoms with history of Asthma with no positive contact history All the above G. None of the above	60	36%
The following can be regarded as respiratory signs and symptoms in the definition of COVID-19		
**Fever Above 37.5** **Cough** **Flue** **Shortness of Breath** Abnormal Respiratory Sound (Wheezes) Itching Eyes and Skin Respiratory symptoms	22	11.6%
A probable cause is.		
**A suspected case for whom testing for COVID-19 is inconclusive.** **A suspected case for whom testing could not be performed for any reason.** A patient with respiratory symptoms and fever with no travel history or contact. All the above None of the above	113	59.8%
A confirmed case for COVID-19 is		
A person with respiratory symptoms and signs plus fever. Positive travel history to high-risk countries/positive contact history **A person with laboratory confirmation of COVID-19 infection/irrespective of clinical signs and symptoms.** All the above None of the above	165	87.3%
When I get a suspected case of COVID-19 at my facility, my immediate response would be		
**Screen and isolate immediately** **Screen for other possible causes of symptoms like malaria, typhoid, tuberculosis, and other possible causes of fever.** Offer first aid and admit to the general ward as I wait for the response team. **Call the response team immediately**	7	3.7%

The correct answers are in bold. The number represents those who got all the correct answers and their percentages. (The definitions were adopted from the Uganda Ministry of Health Guidelines on the management of COVID-19)

### Preparedness of The Health Facilities to Handle COVID-19 Disease

Preparedness of health facilities to handle COVID-19 was assessed by asking 4 questions. These included Ability to admit a patient to Intensive Care Unit, presence of an isolation room at the facility, ability to offer oxygen therapy and ability to offer Intravenous (IV) fluids. Results were as follows:

20/189 (10.5%) of participants could admit a patient to Intensive Care Unit (ICU), 53/189 (28.2%) reported having an isolation space, 50/189 (26.4 %) could Offer IV fluids (Normal saline/ringers), 43/189 (22%) could offer oxygen of about 10L/minute, 23/189 (12.3%) reported that they could not offer any of the above services. The Summary of the findings is summarised in table 2.

**TABLE 2:** Preparedness of the Health Facilities to Handle Emergency Outbreaks

Assessment on the readiness of Health Facilities	N = 189	%
I can admit patients in an intensive care unit (Presence of intensive care unit in a facility)	20	10.5
We have an isolation room (space) at our facility	53	28.2
We can offer IV fluids.	50	26.4
Ability to offer oxygen therapy	43	22
I cannot offer any of the above services	23	12.3

### Availability of Personal Protective Gears (PPEs) and other Materials for Infection prevention and Control (IPC)

This study assessed the availability of materials and equipment used for infection prevention and control. The study assessed the availability of hand washing facilities, availability of protective gears like hand gloves, aprons, and facemasks. The study found that 154/189(81.6%) of the respondents' health facilities had hand washing facilities, 117/189(61.9%) had gloves 92/189, (48.9%) had hand sanitisers, 41/189 (21.6%) had aprons and 72/189 (37.9%) had facemasks.

The study also assessed the availability of protective gears given to patients suspected to be infected with COVID-19. Findings from submitted questionnaires were as follows:

58/189(30.5%) of the participants reported that they could offer gloves and facemasks, 28/189 (14.7%) could offer Facemasks, 24/189(12.6%) could offer Gloves and 76/189 (40%) could neither offer facemasks nor gloves in their facilities.

### Initial Care of a Suspected or Confirmed COVID-19 Case by Front Line Health Workers

Basing on the Uganda clinical Guidelines on management of COVID-19 (First Edition) provided by the Ministry of Health, respondents were asked about their ability to manage mild and severe cases of COVID-19. Outcome of the responses were as follows:

96/189 (50.8%) of the participants knew how to manage mild COVID-19, 50/189(26.5%) knew how to manage-mild COVID-19, 50/189(26.5%) knew how to manage severe cases of COVID-19 and the rest 43/189(22.7%) did not know what to do either for mild case or severe case situation.

**TABLE 3:** Initial Care to Patients with Suspected and Confirmed Case of COVID-19

Regarding management of COVID-19 cases: in mild COVID-19 cases (choose all that apply)	96	50.8%
The patient does not require hospital intervention. Can leave the patient to go back home **Isolation is still necessary to contain the spread of the disease.** Provide a patient with pain killers. Admit patient to the intensive care unit.
Regarding management of severe COVID-19 (choose all that apply)	50	26.5%
**Supplemental oxygen therapy is necessary** Observe patient and offer supportive management knowing the patients' other commodities is not important

### About COVID-19 Continuous Medical Education (CME)/CPD

The study assessed and determined the proportion of health workers who had attended Continuous Medical Education sessions about COVID-19 at their facilities as a way of preparing and re-equipping staff with skills and knowledge to manage COVID-19 cases. 140/189(74.1%) had attended CME workshops while 45(25.9%) had not Attended any.

## DISCUSSION

The ability to detect and identify a probable case of COVID-19 by frontline Health workers is important in ensuring Health workers take immediate appropriate action. This is also important in minimising the spread of the infection and reduction in complications and deaths due to COVID-19.

The current study found that only a small proportion of frontline health workers had good knowledge and understanding of COVID-19 Case management and were able to identify COVID-19 cases by symptom and case definitions as probable case. However, a similar study conducted elsewhere in Uganda reported good knowledge and understanding (80%) of COVID-19 management case.^24^ This is consistent with other studies conducted at the University Hospital in Alexandria, Egypt (81.6%), Dessie referral hospital in Ethiopia (86.4%) and wolaitta sodo hospital(84%).^18–21^

The study also found that a small proportion of health workers 7/189(3.7%) could take appropriate action after identifying a suspected or a probable COVID-19 Case. This is a risky situation since it can easily lead to the spread of the infection among Health workers and to their patients. The study revealed that there is generally minimum level of preparedness by the health facilities to combat COVID-19. This is exhibited through; the inadequate isolation spaces, inadequate IV fluids, and the small number of facilities able to offer oxygen therapy.

These findings were consistent with a Study conducted in Northwest Ethiopia where only 1/8 (12.5%) of the facilities inspected were well prepared to Handle COVID-19 Cases.^21^ The findings are also consistent with findings from another study conducted among hospitals in the Eastern Democratic Republic Of Congo and Western Uganda Hospitals.^22,25^

The study revealed that a good number of health workers had access to Personal Protective Equipment (PPEs) and Infection Prevention and Control (IPC) Measures were put in place at their respective health facilities. That is to say; 154(81.6%) reported having hand washing facilities at their place of work, 92(48.9%) had access to sanitiser and 117(61.9%) had access to gloves However, small percentage 72(37.0%) of health workers reported having face masks. This study finding was consistent with findings in Brazil, Columbia, and Ecuador were a large number of Frontline Health workers reported having inadequate PPEs, gowns and face masks.^26^ The findings were however inconsistent with findings from a study conducted in Eastern DRC and Western Uganda were about 60% of health facilities reported availability of PPEs.^22^ This high level of preparedness is attributed to the fact that the region has experienced multiple outbreaks of Ebola Epidemic and thus the authorities have, with time well equipped its facilities to prepare for any outbreak.

This study has revealed that a good number of health workers can manage mild cases of the pandemic if well facilitated and given support in form of PPEs, IPC materials and provided with the necessary trainings, guidelines, and refresher courses.

## CONCLUSIONS AND RECOMMENDATIONS

There's a need by the Ministry of Health (MoH) through the National and the District task forces or response teams for COVID-19 to plan for and organise mentorship programs in health facilities with emphasis on identification and the immediate actions to be taken in case of suspected, probable, or even confirmed cases for COVID-19 and or any other contagious disease outbreak. Hands-on and simulation training/mentorships is recommended since written guidelines are already in place.

Lower-level Health Facilities should be facilitated and supported in setting up low-cost Case isolation facilities like the use of temporary tents

The Ministry of Health (MoH) and other supporting agencies should provide Facemasks to frontline health workers and health facilities as a priority control measure to the spread of COVID-19 to health workers. Free and affordable control measures like having a hand washing facility at every serving point should be highly encouraged.

Provision of supplies, medical information and carrying out of Continuous Professional Development sessions to frontline Health workers can greatly improve initial patient care and management by frontline health workers and hence minimise cases of cross infections between health workers and patients.
